# Active terahertz beam steering based on mechanical deformation of liquid crystal elastomer metasurface

**DOI:** 10.1038/s41377-022-01046-6

**Published:** 2023-01-04

**Authors:** Xiaolin Zhuang, Wei Zhang, Kemeng Wang, Yangfan Gu, Youwen An, Xueqian Zhang, Jianqiang Gu, Dan Luo, Jiaguang Han, Weili Zhang

**Affiliations:** 1 Center for Terahertz Waves and College of Precision Instrument and Optoelectronics Engineering, Key Laboratory of Optoelectronic Information Technology, Ministry of Education, Tianjin University, 300072 Tianjin, China; 2 Department of Electrical and Electronic Engineering, Southern University of Science and Technology, 518055 Shenzhen, China; 3 School of Electrical and Computer Engineering, Oklahoma State University, Stillwater, OK 74078 USA

**Keywords:** Metamaterials, Photonic devices

## Abstract

Active metasurfaces are emerging as the core of next-generation optical devices with their tunable optical responses and flat-compact topography. Especially for the terahertz band, active metasurfaces have been developed as fascinating devices for optical chopping and compressive sensing imaging. However, performance regulation by changing the dielectric parameters of the integrated functional materials exhibits severe limitations and parasitic losses. Here, we introduce a C-shape-split-ring-based phase discontinuity metasurface with liquid crystal elastomer as the substrate for infrared modulation of terahertz wavefront. Line-focused infrared light is applied to manipulate the deflection of the liquid crystal elastomer substrate, enabling controllable and broadband wavefront steering with a maximum output angle change of 22° at 0.68 THz. Heating as another control method is also investigated and compared with infrared control. We further demonstrate the performance of liquid crystal elastomer metasurface as a beam steerer, frequency modulator, and tunable beam splitter, which are highly desired in terahertz wireless communication and imaging systems. The proposed scheme demonstrates the promising prospects of mechanically deformable metasurfaces, thereby paving the path for the development of reconfigurable metasurfaces.

## Abstract (web summary)

Active terahertz beam steering based on mechanical deformation of the LCE metasurface tuned infrared pump

## Graphical abstract

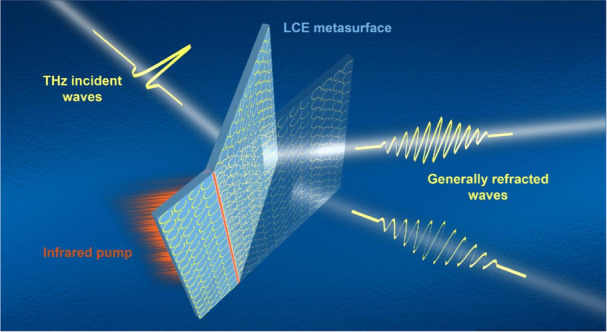

## Introduction

The rich design freedom of subwavelength meta-atoms has enabled unimaginable manipulation of electromagnetic waves. Especially with the introduction of generalized Snell’s law, metasurfaces that can flexibly control the propagation of electromagnetic waves have emerged^1^, which completely avoids the fabrication and loss dilemma of 3D metamaterials. Among the numerous related studies, those tunable ones whose responses can be externally controlled have always been the top priorities. After the pioneering demonstration of the electrically modulated active split-ring resonator (SRR) array in the terahertz band^2^, various methods have been proposed to control the response of meta-atom, ranging from MgH_2_ for the visible light^3^ to the varactor diodes for the microwaves^4,5^. Specific to the terahertz band, the R&D of active metasurfaces is of great significance: on the one hand, active metasurfaces partially alleviate the scarcity of tunable functional devices in the terahertz band. Various terahertz devices have been testified based on active metasurfaces, such as tunable filter^6,7^, optical chopper^8^, compressed sensing imaging^9^, group velocity delay line^10^, and diode-like device^11^. On the other hand, the terahertz dielectric constants of semiconductors^2^, superconductors^7,12^, phase-change materials^13^, 2D materials^11^, and liquid crystals^14–16^ are easily affected by external excitations such as bias voltage, carrier doping, and temperature change, resulting in various modulation methods. However, the reported terahertz active metasurfaces are still far from replacing the traditional devices and being used in terahertz systems on a large scale, because the alteration of the dielectric constant is quite limited. The most widely used method is to control the conductivity of materials in the meta-atoms, such as semiconductors, superconductors, vanadium dioxide, and GST. However, the change of conductivity can only reach the order of 10^5^ S/m in the terahertz band, which is two orders lower than those of metals, causing severe Ohmic loss in performance.

A long-neglected while promising solution to the above problems is the mechanically tunable metasurfaces. Since their tuning originates from mechanical deformation, the dielectric constant remains stable, and no extra dispersion and losses will be introduced. Active metasurfaces based on MEMS^17–21^ have shown great development prospects while their fabrication threshold is relatively high. Remarkably, the recent success of liquid crystal elastomers (LCEs) in soft robotics and optical/thermal-induced mechanical deformation has provided a promising substrate material for mechanically tuning the response of metasurfaces^22–24^. Their deformation may be controlled optically and thermally and has strong reconfigurability, making them useful for various smart devices^25–27^. However, this emerging material has thus far not received extensive attention in the terahertz field. Especially, as a flexible substrate, its potential for regulating the terahertz wavefront is very broad and needs to be developed urgently.

In this paper, we take the lead in demonstrating the wavefront manipulation capability of terahertz metasurfaces based on the mechanical deformation of LCE substrates. The metallic C-shape split-ring resonators (CSRRs) arranged on the LCE substrate are adopted to control the phase and amplitude of the transmitted cross-polarization component based on the generalized Snell’s law. By deflecting and bending the LCE metasurface through infrared illumination and direct heating, active control of the transmitted wavefront was successfully demonstrated by using an angle-resolved terahertz time-domain spectroscopy (THz-TDS) system. Besides, we further investigated the performance and prospect of the proposed LCE metasurfaces as terahertz beam steerer, frequency modulator, and active beam splitter.

## Results

**Fig. 1:**
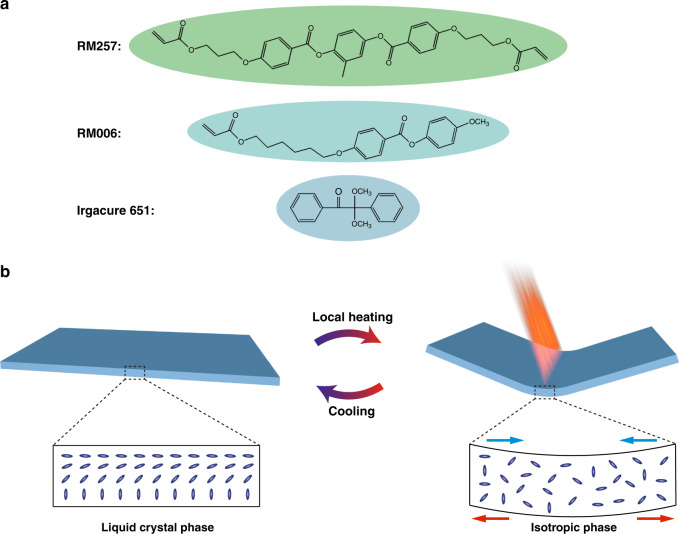
The components of LCE and the mechanism of LCE deformation. **a** Chemical structure of the LCE components. **b** Schematic of the phase-change process of LCE

LCEs are loosely crosslinked polymer networks that can undergo macroscopic deformation when various stimuli (including heat^28,29^, light^30,31^, and electricity^32^) induced phase transition occurs. The LCE substrate is composed of liquid crystal (LC) monomer (RM006), LC crosslinker (RM257), and photoinitiator (Irgacure 651), as shown in Fig. 1a. LCE retains liquid crystallinity and exhibits anisotropic thermal expansion due to the presence of rigid mesogens. Therefore, shape change can be programmed by designing the LCEs’ director field^33,34^. A splayed orientation across the thickness of the LCE substrate is achieved by designing vertical and parallel alignment on different sides of the LC cell. When heated above the nematic-to-isotropic temperature due to the photothermal effect, LCE shrinks on the side with parallel alignment and swells on the other side with vertical alignment. The different strains within a single layer result in a bending action towards the side of parallel orientation, as shown in Fig. 1b.

**Fig. 2:**
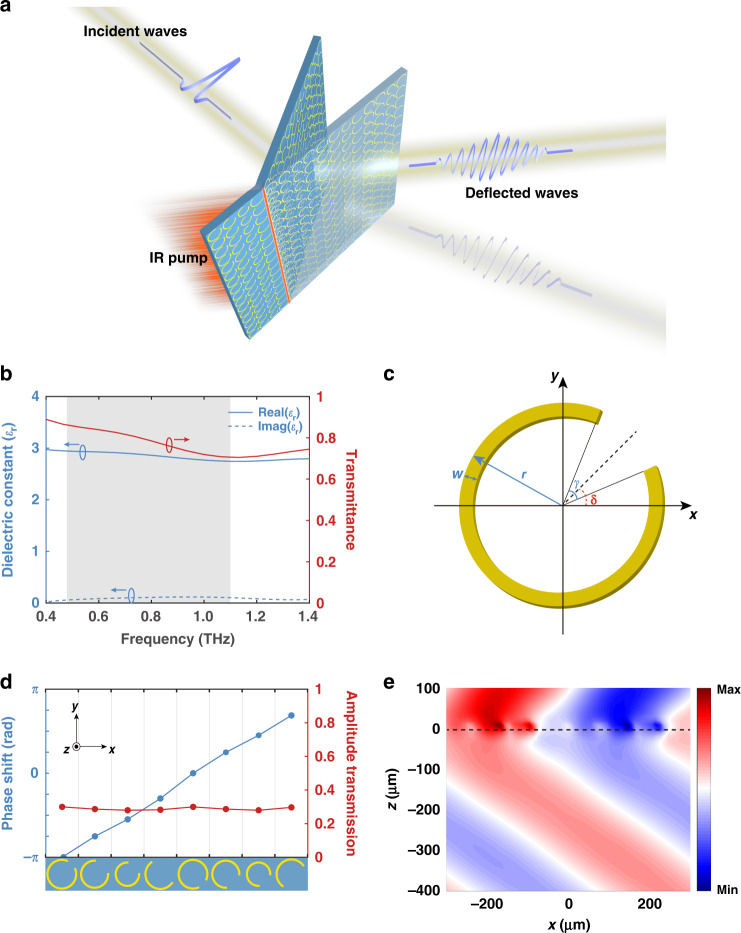
Design of the active LCE metasurface. **a** Illustration of the infrared regulation of the LCE metasurface. **b** The dielectric constant and THz transmittance of LCE in the interested bandwidth. **c** Schematic of the CSRR. **d** The phase shift (blue dot line) and the transmission amplitude (red dot line) of the selected eight CSRRs. **e** Simulated refraction on the designed metasurface at 0.8 THz. The black dashed line indicates the position of the metasurface

To demonstrate terahertz wavefront control based on mechanical deformation, the LCE layer serves as the substrate of a metallic C-shape-split-ring-based phase discontinuity metasurface as illustrated in Fig. 2a. As the beginning of the design process, the 40-μm-thick LCE layer was measured in an 8-F THz time-domain spectroscopy system for retrieving the transmittance and dielectric constant of LCE in the terahertz band. Measured transmission of bare LCE substrate exceeds 84% and the corresponding transmittance is higher than 70%, as shown in Fig. 2b. The real and imaginary parts of the dielectric constant retrieved by the method presented by J. L. Hughes^35^ are shown in Fig. 2b. The calculated refractive index is roughly stable around 1.7, and it can be obtained that the loss of LCE is very small from the tiny imaginary part of dielectric constant. The dichroism and dispersion of LCE are negligible over the entire band of interest, making it an ideal terahertz metasurface substrate.

The selected meta-atoms, i.e., the CSRRs are typical building blocks widely used in representative research of terahertz metasurfaces. When linearly polarized light is incident on the CSRRs, the symmetric and the anti-symmetric modes are excited at the same time^36^, and the phase of the transmitted cross-polarized component can be easily tuned from 0 to 2*π*, so it has absolute advantages to use aluminum CSRRs as unit cells to realize phase discontinuity in the terahertz band as shown in Fig. 2c. By changing the gap width and rotating the gap, the amplitude and phase of the transmitted cross-polarized (*y*-polarized) wave can be independently manipulated (the incident wave is *x*-polarized). The *x*-direction and *y*-direction period of the meta-atom and the linewidth (*w*) of the CSRRs are uniformly set as 640, 80, and 5 μm, respectively. Other parameters of the meta-atoms were determined by sweeping the cross-polarized transmission of CSRRs with varied radius (*r*), gap widths (*γ*), and the orientation of the gap (*δ*) in the CST Microwave Studio^TM^. Finally, eight meta-atoms with nearly equal amplitude were selected to form a superlattice with a 2*π* linear phase gradient and a step of *π*/4, as shown in Fig. 2d. Detailed geometric parameters of these meta-atoms can be found in Table 1. According to the generalized Snell’s law, the periodic arrangement of this superlattice will form a metasurface grating for the transmitted cross-polarized wave. When the *x*-polarized plane wave impinges the metasurface normally, the transmitted *y*-polarized component will maintain the plane wavefront while refracting to the respective angle concerning the frequency, as shown in Fig. 2e (taking 0.8 THz as an example). According to the generalized Snell’s law, the refractive angle for higher frequency is smaller than that for lower frequency. With the selected CSRRs in this work, the output angle under normal incidence ranges from 25° (for 1.1 THz) to 70° (for 0.48 THz).

**Table 1:** Geometric parameters of meta-atoms

	#1	#2	#3	#4	#5	#6	#7	#8
*r* (μm)	37.5	35.5	31	38.5	37.5	35.5	31	38.5
*γ* (deg)	40	80	80	156	40	80	80	156
*δ* (deg)	45	45	45	45	−45	−45	−45	−45

The heading of the column represents the CSRR number. The heading of the row represents the geometry parameters of the CSRRs, including radius (*r*), gap widths (*γ*), and the orientation of the gap (*δ*).

The micron-scale metallic CSRR array on the LCE substrate was fabricated by photolithography and wet etching process (see Supplementary material S1). The mesogens of LCE change from the liquid crystal phase to the isotropic phase when the temperature rises to around 40 °C. Macroscopically, heating results in the deformation of the LCE film in a preset direction, which will change the incident angle, the shape of the CSRRs, and the phase gradient of the metasurface. The study by W. F. Chiang, et al.^37^ showed that even the SRR arrays on a curled LCE substrate cannot be accurately analyzed by theoretical calculation and numerical simulation. More importantly, this bending will change the divergence of the transmitted wavefront in our case. Therefore, here we take the lead in using line-focused infrared light instead of uniformly heating to deflect the LCE metasurface. There is no infrared-sensitive dye in the LCE substrate and LCE deformation is caused by the local heating induced by the infrared pump. To improve the photothermal conversion efficiency, a piece of tinfoil bar with a width of 1 mm and a length of 15 mm was attached to the back of the sample where the infrared light focuses. We found that the tinfoil bar has little effect on the transmission of terahertz waves while can strongly enhance the photothermal effect, benefiting the deflection of LCE metasurface with much lower infrared pump power. The refraction of the sample was measured in a self-made angle-resolved THz-TDS system (see the “Materials and methods” section at the end of text). In the experiment, the infrared light was precisely line-focused onto the tinfoil bar, which raised the local temperature of the irradiated area. The heated LCE bent due to temperature rise while other areas of the substrate were kept flat. When the pump power of line-focused infrared light reaches 200 mW, the LCE starts to deflect with the spot line as the revolving axis. More importantly, the unilluminated part remains perfectly flat (see Supplementary video 1). As the optical power increases, the deflection angle increases accordingly. The highest average power applied was 400 mW, and the maximum deflection angle achieves 18°. The average power used in this work has reached the order of 100 mW, which is mainly caused by the high transparency of LCE to 1030 nm infrared light. By changing the pump wavelength or doping absorbing components such as MXene, the average power of the pump can be effectively reduced to the order of tens or several milliwatts.

**Fig. 3:**
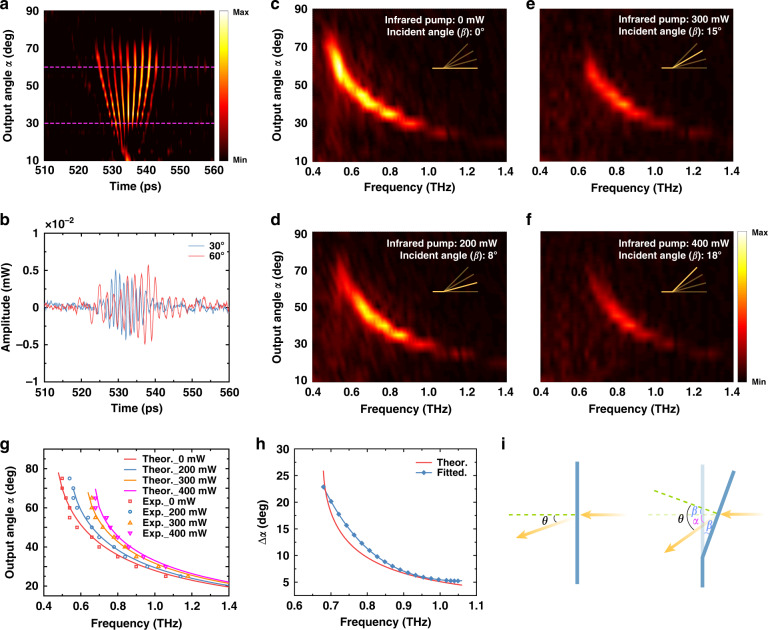
Infrared regulation of the LCE sample. **a** Received time-domain signals at varied output angles. **b** Time-domain signals received at output angles of 30° and 60°. **c–f** Fourier transform of the received time-domain signals under 0, 200, 300, and 400 mW infrared pump. The insets indicate the deflection of the LCE sample. **g** The frequency-resolved output angles at each pump power. The solid lines show the output angles predicted by the generalized Snell’s law and the hollow square, circle, and up-and-down triangles are the measured ones. **h** Output angle variations (Δ*α*) at different frequencies between no pump and 400 mW pump. The red line was theoretically calculated, and the blue dotted line is the fitted results of the measured data. **i** Influence of the LCE substrate deflection on the refraction of terahertz waves

The investigation of the tuning ability of the LCE metasurface begins with the no-pump case. Figure 3a lists the *y*-polarized time-domain signals through the sample at various output angles and Fig. 3b shows the time-domain signals at output angles of 30° and 60° as examples. The *y*-polarized component is refracted to different output angles, which accords with our prediction and previous studies^36^. In the next step, the infrared pump was applied, and the pump power was set to 200, 300, and 400 mW, resulting in the LCE deflection angles of 8°, 15°, and 18°, respectively. At each pump power, the *y*-polarized time-domain signals of the deflected LCE sample were measured repeatedly. It should be noted that although the maximum power reaches 400 mW, the infrared pump does not denature the substrate. When the infrared light was removed, the LCE metasurface returns to the no-pump state within 5 s. Fourier transform was then applied to the measured time-domain signals and the frequency-resolved output angles are depicted in Fig. 3c–f. First, for each pump case, the LCE sample serves as a cross-polarization grating quite well, in which a narrow-band *y*-polarized plane wave is refracted to a specific output angle according to the center frequency of the wave. The second point drawn from Fig. 3c–f is that the efficiency of the LCE metasurface reduces as the infrared pump increases, resulting in a useful frequency band ranging from 0.48 to 1.1 THz as shown in Fig. 3g. Referring to the most interesting wavefront steering capability, we depict the center frequencies at each measured output angles in Fig. 3g. The infrared pump obviously changes the output angle of the transmitted *y*-polarized wave and the frequency dependence of the output angle. The change of the output angle, i.e., the wavefront steering is more obvious as the pump power increases from 0 to 400 mW. As summarized in Fig. 3h, for 0.68 THz, a maximum output angle tuning of 22° is experimentally achieved. This demonstrates considerable advantages in steering angle and tuning capability compared to other beam-steering metasurfaces. For example, the metasurface of using phase change material GST to achieve beam deflection can only be switched back and forth along the designed steering angles and cannot be continuously controlled^38^. Though this wavefront steering ability gradually shrinks as frequency increases, a tuning range of 5° was still demonstrated at 1.1 THz (the highest frequency in the interested spectral band).

The above-mentioned infrared regulation of the LCE metasurface can be explained by combining the thermomechanical deformation of the LCE substrate and the generalized Snell’s law. The infrared pump light heats the illuminated part, resulting in the deflection of the LCE substrate around the line-focused position. Since the unirradiated area of the substrate remains flat, the infrared pump introduces tilted incidence on the LCE metasurface as shown in Fig. 3i. We checked each CSRR under titled incidence up to 18° and found that the cross-polarized transmission phase is quite robust to tilted incidence. With this in hand, we use the generalized Snell’s law to calculate the frequency-resolved output angle under oblique incidence, which can be expressed as

1 $$\alpha = \theta - \beta = {{{\mathrm{arcsin}}}}\left(\sin \beta + \frac{c}{{2{\uppi}f}}\frac{{{{{\mathrm{d}}}}\Phi }}{{{{{\mathrm{d}}}}x}}\right) - \beta$$

where *α*, *θ*, and *β* are the output angle, refractive angle, and deflection angle, respectively. *c* and *f* are the light velocity in vacuum and frequency, respectively. Φ is the cross-polarized transmission phase and dΦ/d*x* is the phase gradient formed by the CSRR superlattice. The calculated frequency dependence of the output angle was depicted as the lines in Fig. 3g, h, which are in good accordance with the measured results for each pump case. Minor discrepancies may come from the deflection-caused spatial translation of the LCE substrate. Equation 1 provides a convenient way to understand the infrared regulation of this LCE metasurface. For example, by calculating ∂*α*/∂*β* and ∂*α*/∂*f*, it can be deduced that (1) the regulation capability for low frequency is sounder than that for high frequency and (2) the change of output angle is more sensitive for low pump power. These conclusions are also well demonstrated by the measurement. Referring to the decrease of the cross-polarized amplitude with the LCE deflecting mainly comes from the emergence of other diffraction orders as the incidence angle increases. As an effective diffraction grating, the increment of the incident angle will produce other diffraction orders in the non-detection area, such as 0th, −1st, and −2nd order. These new diffraction orders will take away part of the energy, reducing the amplitude of the +1st order. The larger the incident angle is, the more the amplitude decreases, which is consistent with that shown in Fig. 3c–f.

**Fig. 4:**
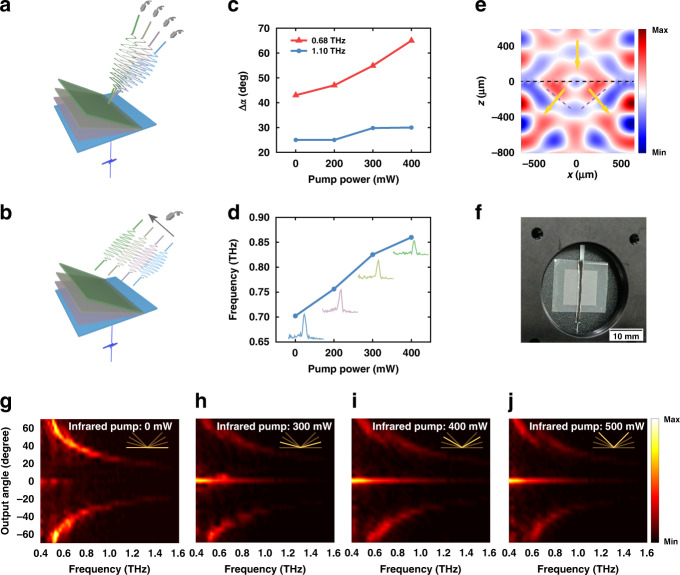
Application investigation of LCE metasurfaces. **a** Schematic of the LCE metasurface as a terahertz beam steerer controlled by infrared light. **b** Schematic of the LCE metasurface as a terahertz frequency modulator when the detection is fixed at an output angle of 40°. **c** Variations of the output angle for 0.68 and 1.1 THz when pump power increases from 0 to 400 mW. **d** Detected center frequencies versus the pump power at 40° output angle. **e** Simulated beam splitting under normal incidence for 0.8 THz. **f** Photo of the fabricated LCE beam splitter suspended on the holder. The thick black line in the middle indicates where the infrared light impinges. **g–j** Frequency-resolved beam splitting behavior under different pump power. The insets indicate the deflection of the splitter

The infrared regulation of the LCE metasurface makes it promise to be developed into a variety of functional devices with practical needs in the terahertz band. The most obvious potential application of such active metasurfaces is for beam steering in next-generation wireless communications. As illustrated in Fig. 4a, the variation of output angle makes the LCE metasurface an excellent beam steerer controlled by an external infrared pump. As shown in Fig. 4c, as the pump power increases from 0 to 400 mW, the output beam of 0.68 THz was steered from 43° to 65° with quite a good linearity, realizing a tuning range of 22°. For higher frequencies, this steering ability gradually shrinks. At 1.1 THz the tuning ability is limited to 5°. On the other hand, if the terahertz detector was fixed at 40°, this LCE metasurface works as a practical frequency modulator as illustrated in Fig. 4b. By altering the pump power, the center frequency of the received terahertz signals ranges from 0.70 to 0.86 THz as shown in Fig. 4d. And as shown in the inset, the linewidths of the received signals under a specified pump power are <0.12 THz.

Besides beam steering and frequency shifting, the LCE metasurface grating can be developed as a novel active beam splitter as pointed out in our previous researches^39,40^. We placed two CSRR gratings mentioned above on the same LCE substrate and made the phase gradients of the two gratings mirror symmetrical. When the *x*-polarized beam imping onto the metasurface, the transmitted *y*-polarized component will be split into two separated beams, as shown in Fig. 4e. As discussed by M.G. Wei^39^ and R.D. Jia^40^, the splitting ratio can be easily controlled by translating the incident beam thus re-dividing the area on each grating. More importantly, the output angles of the two split beams can be manipulated by adding a line-focused infrared pump on the seam. In the measurement, the fabricated LCE beam splitter was suspended along the splice by a thin wire pasted on the back of the substrate, as shown in Fig. 4f. In the active manipulation of the metasurface, the infrared light was focused onto the splice. The measured beam splitting effect is shown in Fig. 4g–j, where the frequency-resolved deflection angles from both sides have changed significantly with the alteration of pump power. As for the output at 0°, when there is no infrared pump as shown in Fig. 4g, it is due to the diameter of the incident terahertz beam being slightly larger than the sample. Thus, a very small fraction of the terahertz wave will bypass the sample and be received directly by the detector. Although the detector is cross-polarized, a residual signal can still be detected due to the incomplete linear polarization of the incidence and the limited extinction ratio of the terahertz polarizers. When the LCE splitter was deflected, the output at 0° mainly comes from two sources. First, the folding of the sample narrows the cross-section of the sample, causing more incident terahertz wave bypasses the sample. And the intensity of this signal is obviously proportional to the deflection angle (*β*). Second, the increase of the incidence angle caused by the deformation of the sample will lead to the 0th diffraction order in addition to the main diffraction order (+1st), which contributes to the output at 0°. As the deflection angle (*β*) increases, the amplitude of the 0th diffraction order increases gradually as well, in accordance with Fig. 4h–j.

## Discussion

**Fig. 5:**
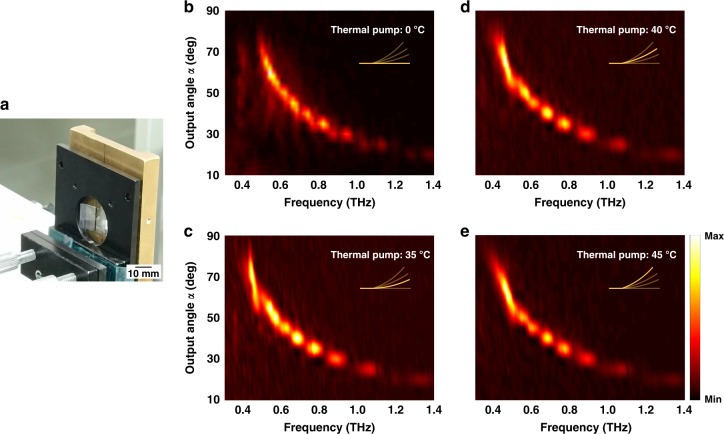
Thermal regulation of the LCE metasurface. **a** The photo of the mounted LCE deflector and hot plate. **b–e** Frequency-resolved output angles of the LCE metasurface under 22 °C room temperature, 35, 40, and 45 °C. The insets illustrate the curling of the LCE metasurface

Since LCE deformation is caused by temperature change, we also conducted an active control study using direct heating as another manipulation method. In the measurement, a hollow heating plate was placed 1 mm in front of the sample holder, and terahertz waves passed through the rectangular hole in the heating plate. By raising the temperature of the heating plate, the nearby LCE substrate was uniformly baked to curl as shown in Fig. 5a. Figure 5b–e shows the frequency dependence of the output angles (*y*-polarized) at 22 °C room temperature, 35, 40, and 45 °C (measured on the heating plate), with the inset showing the curling of the LCE metasurface. The discontinuities in Fig. 5b–e is due to the angle interval of the scanning. During the measurement, the deflected wave was detected at an angle resolution of 5° by rotating the rotation stage. Since the higher frequency is more sensitive to the change of the output angle (*α*), the frequency-angle resolved output at high frequency exhibits more serious discontinuities. Unlike the line-focused infrared pump, uniform heating bends the whole LCE metasurface, resulting in changes in the outgoing direction of the cross-polarized component. Because the phase gradient of the meta-atom superlattice is no longer linear for the curled metasurface, it is foreseeable that the transmitted cross-polarized wavefront will not remain a plane wave, which is an essential difference compared to the line-focused infrared regulation. Though heating shows different manipulation feature, the controllable curling of LCE metasurface may result in focus tunable metalens, and the thermal control of LCE metasurfaces are widely promising for thermal-induced deformation application scenarios such as smart wearable devices working at terahertz frequencies.

Besides the deformation ability shown above, modulation speed is an important feature for active metasurface as well. In our work, the modulation speed is determined by the deflection and recovery speed of the LCE substrate. Both are in the order of seconds, because of the limited photothermal conversion speed and the relatively large size of the sample. However, improving the modulation speed is possible by adopting appropriate metasurface designs. We initially propose two methods. One is to pixelate the deformation part instead of bending the entire sample. We expect a higher modulation speed can be achieved when the size of the deformation part is reduced. The other is to add a photosensitive component to the LCE material to replace photothermal control. The traditional way is to realize the transformation of azobenzene isomers induced by light^41^, other possible method includes using the light-induced reversible transformation of chiral groups^42,43^, etc. Once these modulation methods are applied to LCE, the modulation speed will be greatly improved and the research value and application prospect of the mechanical deformation-based metasurface will be greatly expanded.

In summary, we have designed a phase discontinuity metasurface with LCE as substrate and C-shape split-rings as resonators for controllable broadband terahertz wavefront steering. From 0.48 to 1.1 THz, the LCE metasurface steers the cross-polarized transmission beam from 70° to 25°. Based on the deformation response characteristics of LCE, we applied an infrared laser pump and direct heating to realize the deflection of the metasurface. An output angular increment of 22° was achieved at 0.68 THz under 400 mW infrared light pump. We also presented the promising potential of LCE metasurface as a beam steerer, frequency modulator, and tunable beam splitter. The LCE metasurface demonstrated here lays the foundation for the design of mechanical deformation-based metasurface and has broad prospects in next-generation high-speed terahertz wireless communication and advanced T-ray imaging systems.

## Materials and methods

In our experiment, the LCE substrate was composed of LC monomer (RM006), LC crosslinker (RM257), and photoinitiator (Irgacure 651). The weight ratio of RM006, RM257, and Irgacure 651 was 49.5:49.5:1. The chemical structures of the LCE are shown in Fig. 1a.

**Fig. 6:**
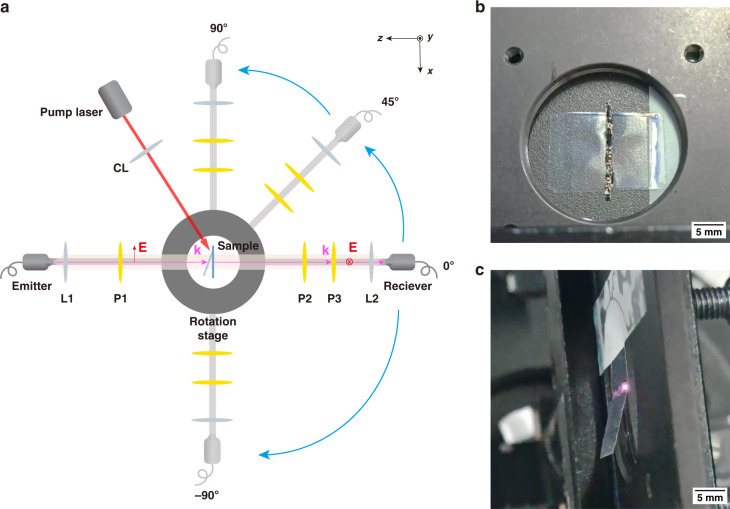
The experiment setup for testing the infrared regulation of the LCE metasurface. **a** The schematic of the measurement optical system. L: Lens, CL: Cylindrical lens, and P: Polarizer. The deflected wave was detected at an angle resolution of 5° by rotating the rotation stage. **b** The sample holder. **c** the deflection of the LCE sample when 400 mW infrared light was focused on the sample

As for the experimental section, the sample was measured in a THz-TDS system as illustrated in Fig. 6a: a fiber-coupled horizontal-polarized (*x*-polarized) THz transmitter followed by a lens and a horizontally orientated polarizer deliver a collimated and *x*-polarized THz beam with a diameter of 5 mm. The sample holder is placed 17.5 cm away from the lens and normally to the incident THz beam, as shown in Fig. 6b. THz wave refracted by the metasurface passes two vertically orientated (*y*-polarized) THz polarizers for completely filtering out the *x*-polarized component. Then the *y*-polarized wave is focused by another lens and detected by a fiber-coupled *y*-polarized THz receiver. The last two polarizers and the detection part are all mounted on a guide that can revolve automatedly around the sample holder. In the measurement, this guide rotates to a specific output angle and the refracted cross-polarized terahertz time domain signal is sampled. When the sampling is accomplished, the guide rotates to the next output angle and the signal sampling process is repeated. The THz-TDS system is based on asynchronous sampling with a scan rate of 50 Hz and a 500-times average is applied for an acceptable SNR.

We chose an amplifier that generates high-energy pulses with 1030 nm wavelength and 260 fs pulse width by chirped pulse amplification, which can deliver an average power of up to tens of watts. In addition, the output power of the laser can be precisely adjusted by a pulse picker to investigate the relation between the LCE deformation and the pump power. By using a cylindrical lens, the infrared pump was focused at one side of the CSRR array as a line along the *y*-axis with a width of 1 mm and a length of 15 mm, as shown in Fig. 6c. The single pulse energy was set as 200 μJ and the average power illuminated on the LCE substrate was changed by adjusting the repetition frequency.

## Supplementary information

Supplementary Information for Active Terahertz Beam Steering Based on Mechanical Deformation of Liquid Crystal Elastomer Metasurface

Supplementary Video 1

## Data Availability

All data are available from the corresponding authors upon reasonable request.
