# Supplementary material for: Active terahertz beam steering based on mechanical deformation of liquid crystal elastomer metasurface
Source: Light Sci Appl. 2023 Jan 4;12:14. doi: 10.1038/s41377-022-01046-6 (PMC9810742; doi:10.1038/s41377-022-01046-6)
Supplement: Supplementary file 1 — Supplementary Information for Active Terahertz Beam Steering Based on Mechanical Deformation of Liquid Crystal Elastomer Metasurface [file 41377_2022_1046_MOESM1_ESM.docx]

**Supplementary Information for**

**Active Terahertz Beam Steering Based on Mechanical Deformation of Liquid Crystal Elastomer Metasurface**

**Xiaolin Zhuang^1^**^†^**, Wei Zhang^2^**^†^**, Kemeng Wang^1^, Yangfan Gu^1^, Youwen An^1^, Xueqian Zhang^1^, Jianqiang Gu^1*^, Dan Luo^2*^, Jiaguang Han^1^ and Weili Zhang^3^**

*^1^Center for Terahertz Waves and College of Precision Instrument and Optoelectronics Engineering, Key Laboratory of Optoelectronic Information Technology, Ministry of Education of China, Tianjin University, Tianjin 300072, China*

*^2^Department of Electrical and Electronic Engineering, Southern University of Science and Technology, Shenzhen 518055, China*

*^3^School of Electrical and Computer Engineering, Oklahoma State University, Stillwater, OK 74078, USA*

*^*^Corresponding author: gjq@tju.edu.cn , luod@sustech.edu.cn*

*^†^These authors contributed equally to this work*

**S1 Fabrication of sample**

The micron-scale metallic CSRR array on the LCE substrate was fabricated by photolithography and wet etching process, for avoiding the irrecoverable pleats on the LCE substrate during the thermal deposition as well as the curl or dissolve caused by the long-time soaking in the lift-off solution. The detailed processing flow of the fabrication is shown in Fig. S1a. First, a uniform aluminum layer with a thickness of 200 nm was evaporated on the blank LCE film by controlling the speed of thermal evaporation which avoided any bending of the LCE film and maintains good metallic layer adhesion. After that, a complementary photoresist pattern was transferred onto the aluminum layer by photolithography. Then the sample is soaked in the aluminum etching solution for 5 minutes and rinsed to remove excess aluminum. At last, the residue photoresist was removed by soaking in the developer solution. The global and microscopic graphs of the fabricated LCE metasurface sample are shown in Fig. S1b, where the sample is flexible and quite flat, and the CSRR meta-atoms are in precise shape. The measured minimum linewidth is 5 μm, which fully meets the design.


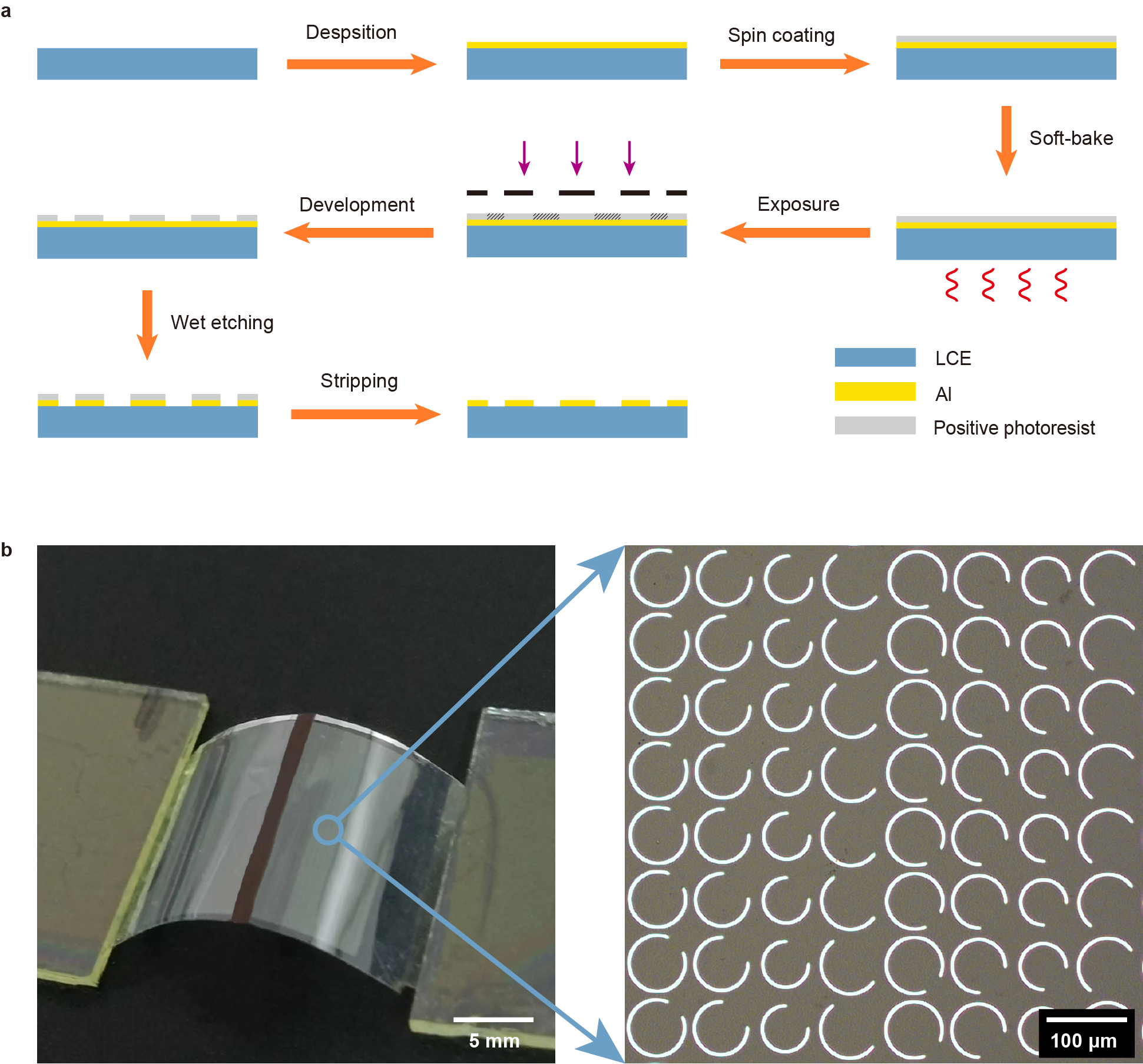


**Fig. S1 Fabrication of the LCE metasurface sample. a** Detailed fabrication process of the LCE metasurface sample. **b** The flexible LCE sample and the microscopic image of the CSRR meta-atoms. The thick black line in the middle of the LCE sample indicates the illumination position of the line-focused infrared light.

**Supplementary video 1:**

The sample is deflected upward with the spot line as the revolving axis by infrared pump, and the unilluminated part remains perfect flat.
